# Mapping the Genetic Architecture of Gene Regulation in Whole Blood

**DOI:** 10.1371/journal.pone.0093844

**Published:** 2014-04-16

**Authors:** Katharina Schramm, Carola Marzi, Claudia Schurmann, Maren Carstensen, Eva Reinmaa, Reiner Biffar, Gertrud Eckstein, Christian Gieger, Hans-Jörgen Grabe, Georg Homuth, Gabriele Kastenmüller, Reedik Mägi, Andres Metspalu, Evelin Mihailov, Annette Peters, Astrid Petersmann, Michael Roden, Konstantin Strauch, Karsten Suhre, Alexander Teumer, Uwe Völker, Henry Völzke, Rui Wang-Sattler, Melanie Waldenberger, Thomas Meitinger, Thomas Illig, Christian Herder, Harald Grallert, Holger Prokisch

**Affiliations:** 1 Institute of Human Genetics, Helmholtz Center Munich, German Research Center for Environmental Health, Neuherberg, Germany; 2 Institute of Human Genetics, Technical University Munich, München, Germany; 3 Research Unit of Molecular Epidemiology, Helmholtz Center Munich, German Research Center for Environmental Health, Neuherberg, Germany; 4 Institute of Epidemiology II, Helmholtz Center Munich, German Research Center for Environmental Health, Neuherberg, Germany; 5 German Center for Diabetes Research (DZD e.V.), Neuherberg, Germany; 6 Interfaculty Institute for Genetics and Functional Genomics, Department of Functional Genomics, University Medicine Greifswald, Greifswald, Germany; 7 Institute for Clinical Diabetology, German Diabetes Center, Leibniz Center for Diabetes Research at Heinrich Heine University Düsseldorf, Düsseldorf, Germany; 8 German Center for Diabetes Research (DZD e.V.), partner site Düsseldorf, Germany; 9 Institute of Molecular and Cell Biology, University of Tartu, Tartu, Estonia; 10 Estonian Genome Center, University of Tartu, Tartu, Estonia; 11 Department of Prosthetic Dentistry, Gerostomatology and Dental Materials, University Medicine Greifswald, Greifswald, Germany; 12 Institute of Genetic Epidemiology, Helmholtz Center Munich, German Research Center for Environmental Health, Neuherberg, Germany; 13 Department of Psychiatry and Psychotherapy, Helios Hospital Stralsund, University Medicine of Greifswald, Greifswald, Germany; 14 Institute of Bioinformatics and Systems Biology, Helmholtz Center Munich, German Research Center for Environmental Health, Neuherberg, Germany; 15 Estonian Biocentre, Tartu, Estonia; 16 Institute of Clinical Chemistry and Laboratory Medicine, Greifswald, Germany; 17 Division of Endocrinology and Diabetology, University Hospital Düsseldorf, Düsseldorf, Germany; 18 Institute of Medical Informatics, Biometry and Epidemiology, Ludwig-Maximilians-Universität Munich, Neuherberg, Germany; 19 Department of Physiology and Biophysics, Weill Cornell Medical College in Qatar, Education City, Qatar Foundation, Doha, Qatar; 20 Institute for Community Medicine, University Medicine Greifswald, Greifswald, Germany; 21 Munich Heart Alliance, München, Germany; 22 Hannover Unified Biobank, Hannover Medical School, Hannover, Germany; Queen's University Belfast, United Kingdom

## Abstract

**Background:**

We aimed to assess whether whole blood expression quantitative trait loci (eQTLs) with effects in *cis* and *trans* are robust and can be used to identify regulatory pathways affecting disease susceptibility.

**Materials and Methods:**

We performed whole-genome eQTL analyses in 890 participants of the KORA F4 study and in two independent replication samples (SHIP-TREND, N = 976 and EGCUT, N = 842) using linear regression models and Bonferroni correction.

**Results:**

In the KORA F4 study, 4,116 *cis*-eQTLs (defined as SNP-probe pairs where the SNP is located within a 500 kb window around the transcription unit) and 94 *trans*-eQTLs reached genome-wide significance and overall 91% (92% of *cis-*, 84% of *trans-*eQTLs) were confirmed in at least one of the two replication studies. Different study designs including distinct laboratory reagents (PAXgene™ vs. Tempus™ tubes) did not affect reproducibility (separate overall replication overlap: 78% and 82%). Immune response pathways were enriched in cis- and trans-eQTLs and significant cis-eQTLs were partly coexistent in other tissues (cross-tissue similarity 40–70%). Furthermore, four chromosomal regions displayed simultaneous impact on multiple gene expression levels in trans, and 746 eQTL-SNPs have been previously reported to have clinical relevance. We demonstrated cross-associations between eQTL-SNPs, gene expression levels in trans, and clinical phenotypes as well as a link between eQTLs and human metabolic traits via modification of gene regulation in cis.

**Conclusions:**

Our data suggest that whole blood is a robust tissue for eQTL analysis and may be used both for biomarker studies and to enhance our understanding of molecular mechanisms underlying gene-disease associations.

## Introduction

The key aim of human genetics is to elucidate molecular mechanisms underlying phenotypic variation, particularly with respect to disease and disease susceptibility [1]. In recent years, genome-wide association studies (GWAS) have successfully tagged more than three thousand disease or trait associated genetic loci. However, often, molecular mechanisms linking the locus to the disease phenotype remained unclear. Analysis of whole genome expression quantitative trait loci (eQTL) provide a means for detecting transcriptional regulatory relationships at a genome-wide scale and thus for identifying regulatory pathways affecting disease susceptibility [2].

For the analysis of mechanisms of gene regulation, the question of tissue dependency and specificity is of major relevance. In particular, expression patterns of disease-relevant tissues might further advance our understanding of disease pathophysiology. However, for ethical and practical reasons, eQTL analyses are often only feasible in whole blood rather than in disease-relevant tissues, particularly in large population based observational studies. Therefore, it is important to assess, whether regulation of gene expression in whole blood is robust and reflects gene expression patterns in other tissues or cell lines. In the past, eQTL mapping studies in whole blood have focused on genetic variation with an impact on gene regulation acting in *trans* to identify down-stream mechanisms of these variants on clinical phenotypes or were restricted in sample size [2]–[4].

In a previous work conducted in 322 European subjects, we found that whole blood eQTLs with effects in *cis* were reproducible, while the power to address this question for eQTLs with effects in *trans* was limited due to the burden of multiple testing [4]. For that reason, here, we used a larger, distinct discovery sample and investigated data of 890 participants of the Cooperative Health Research in the Region of Augsburg (KORA) F4 and 1,818 participants of two replication samples.

Furthermore, up to now, it is unclear whether the study design or technical issues such as time of blood collection or usage of distinct laboratory tools have an impact on the reproducibility of results. These factors might be important as for example gene expression profiles in whole blood cell samples obtained using different laboratory reagents (PAXgene™ and Tempus™ blood collection tubes) and protocols had been found to differ to large extents with the result of recommendations not to use them for joint or meta-analysis [5]. In our recent work, cis-eQTLs in whole blood were reproducible under the same study conditions. In the present work, we examine whether eQTLs in both cis and trans are reproducible even if they are obtained under different study conditions by validating results of the discovery analysis in two independent European replication samples, the Study of Health in Pomerania (SHIP) TREND study and the Estonian Biobank (Estonian Genome Center, University of Tartu (EGCUT)) study. One of these samples (SHIP TREND) was designed similarly to the KORA F4 discovery study, while the other (EGCUT) displayed multiple differences in the study design including distinct laboratory reagents and protocols (details are described in Table 1).

**Table 1:** Study description of KORA F4, SHIP-TREND and EGCUT.

	N	Age (years)	Sex	Fasting status	RNA collection	RNA isolation	Expression Chip	Genotyping	Imputation
KORA F4	890	70.57±5.42	448 males, 442 females	fasting (8 non-fasting samples)	PAX tubes	PAXgene Blood miRNA Kit	Illumina HumanHT-12 v3	Affymetrix 6.0	
SHIP-TREND	976	50.12±13.74	428 males, 548 females	all fasting	PAX tubes	PAXgene Blood miRNA Kit	Illumina HumanHT-12 v3	Illumina HumanOmni2.5-Quad	IMPUTE v2.1.2.3
EGCUT	842	37.16±15.60	415 males, 427 females	non-fasting	Tempus tubes	Tempus Spin RNA Isolation Kit	Illumina HumanHT-12 v3	Illumina Human370CNV	IMPUTE v2.2.3

In addition to this, results were compared to publicly available *cis-*eQTLs found in other tissues or cell line studies in order to assess whether whole blood eQTLs are tissue specific or shared across different tissues and cell lines.

Moreover, we applied pathway analysis to detect functional properties of transcriptional regulatory relationships in whole blood. Finally, we (i) identified eQTLs with simultaneous impact on multiple gene expression levels, (ii) compared significant *cis-*eQTLs to published results of GWA studies, and (iii) compared eQTLs to metabolomic quantitative trait loci (metQTLs) found previously in the KORA F4 study to provide examples of how whole blood eQTL studies might contribute to our understanding of molecular mechanisms determining phenotypes.

## Results and Discussion

### Robustness of whole blood eQTLs

#### a) Discovery and replication of eQTLs

The present eQTL study comprised data of 2,708 subjects (890 subjects of the KORA F4 discovery sample and 1,818 subjects of two independent replication samples). Thus, it is one of the largest genome-wide eQTL study analyzing both effects in *cis* and in *trans* in whole blood cell samples from European populations so far.

Altogether, 4,210 eQTLs reached genome-wide significance (6.02E-9 and 2.81E-12 for *cis-* and *trans-*eQTL, respectively) in linear regression analysis using the conservative Bonferroni approach as correction method for multiple testing to prevent false-positive findings.

**Figure 1:**
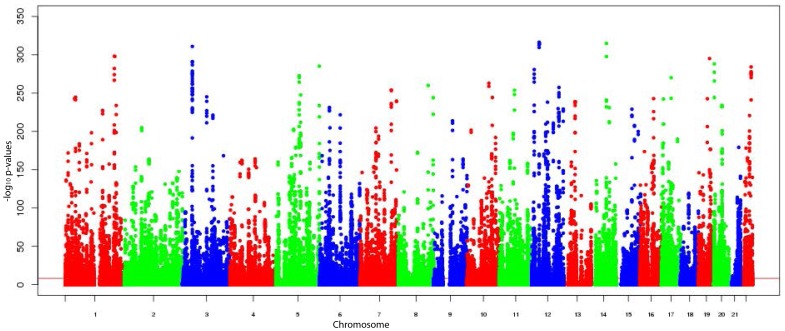
Manhattan plot of all analyzed *cis-*eQTLs with their calculated p-values in the KORA F4 study.

**Figure 2:**
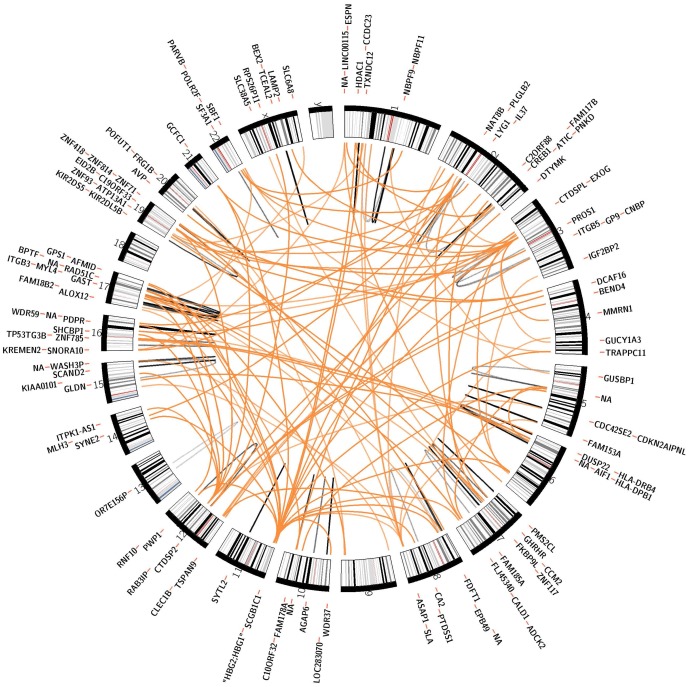
All *trans*-eQTLs with p-values below 2.81E-12 in the KORA F4 study.

Among the identified eQTLs, 4,116 eQTLs resided within in a 500 kilobase (kb) window around the transcription start and end site of the affected gene and thus, were defined *cis-*eQTLs (P-value range  = 6.1E-299 to 6.0E-9, Figure 1). The 4,116 *cis-*eQTLs corresponded to 3,449 RefSeq genes (HG19); for 515 *cis*-eQTLs a signal with two or more different transcript probes was observed while 79 *cis-*eQTLs could not be assigned to a specific gene. The remaining 161 SNP-probe associations were defined as *trans-*eQTLs. After pruning SNPs being in high linkage disequilibrium (LD), of the 161 SNP-probe associations 94 genomic loci with an impact on more distant genes remained (P-value range  = 2.8E-248 to 2.8E-12, Figure 2).

Of all significant eQTLs, 3,847 eQTLs making up 91% of all detected eQTLs (92% of *cis*-, 84% of *trans-*eQTLs) were replicated in at least one of the two independent replication samples SHIP-TREND (N = 976) and EGCUT (N = 842, Bonferroni corrected p-values  =  1.21E-5 and 3.1E-4 for *cis-* and *trans-*eQTLs, respectively). For congruent SNP-probe associations we calculated 99% consistency in allelic directions indicating a high reliability of results. The set of confirmed significant *trans*-eQTLs included 144 SNP-probe associations representing 79 genomic loci and the set of confirmed significant *cis*-eQTLs comprised 3,768 SNPs corresponding to 3,176 RefSeq genes (Table S1 and S2).

#### b) Reproducibility of eQTLs

Previously, it had been reported that gene expression profiles in whole blood samples obtained using different laboratory reagents (PAXgene™ and Tempus™ blood collection tubes) and protocols differ to large extents and, therefore, the authors recommended that results across studies derived from different RNA collection tubes should not be compared [5]. In order to investigate to which extent these systematic differences are also affecting whole blood eQTL studies, we aimed to replicate our results in two independent samples, one sample, SHIP-TREND, using a similar study design as the KORA F4 discovery sample and the other, EGCUT, displaying multiple differences in the study design and sample preparation. These differences were related to the fasting status of participants (participants of the KORA F4 and SHIP-TREND study fasted for at least eight hours prior to blood donation whereas those of the EGCUT study did not fast), differences in time of blood collection (Figure S1), and the usage of distinct laboratory reagents (PAXgene™ tubes were used in the KORA and SHIP-TREND studies and Tempus™ tubes in the EGCUT study). In spite of these differences, p-values of the three studies were largely congruent (Figure S2). Furthermore, the overlap of replication in both samples was similar (82% in EGCUT, 85% of cis- and 72% of trans-eQTLs, respectively and 78% in SHIP-TREND, 81% of cis- and 75% of trans-eQTLs). This demonstrates that, in contrast to whole blood gene expression profiles, whole blood eQTLs are highly robust.

#### c) Cell-type specificity of whole blood eQTLs

Gene expression patterns vary among different cell types. Because whole blood cells provide the most convenient tissue for analysis, it is of relevance whether it is a suitable surrogate tissue and reflects transcriptional relationships also in other tissues. Therefore, one of our study aims was to investigate whether our results are comparable to those obtained in cell lines and tissues other than whole blood. Unfortunately, databases provided by other studies were publicly available only for *cis-*eQTLs [2], [3], [6]–[12]. Comparing our results to those listed in these databases we observed reasonable concordance of 65%–68% with two more *cis*-eQTL studies conducted in whole blood and 51%–70% with *cis*-eQTL studies conducted in primary monocytes or blood-derived lymphoblastoid cell lines (LCLs) (Table 2). Furthermore, in line with two additional studies comparing eQTLs in LCLs and fibroblasts [13] and in LCL, skin, and fat [14], we observed major cross-tissue similarity when comparing our results to those of eQTL studies conducted in B cells, lung and liver tissue (40–70%, Table 2). In contrast, two of the first eQTL studies conducted in small population samples found that the genetic control mechanisms of gene expression in whole blood and LCLs as well as in primary fibroblasts, LCLs and T cells are largely independent [3], [15], [16]. This contradictory finding might be attributable to low statistical power. Thus, it evokes the hypothesis that those effects which are shared across different tissues and cell lines might be smaller than tissue- and cell-specific effects or more heterogeneous (i.e. the effect being small in just one cell type). Consequently, cross-tissue similarity might prove to be even higher in yet larger study samples. Furthermore it should be noted that the studies had different power to detect true eQTLs which might also result in an underestimated overlap between tissues [13]. More precise methods for estimating the overlap could have been used if either raw data of all studies were available [17] or effect sizes across studies were comparable [13], i.e. by using the same expression array types. Although some of the eQTL studies which were used for comparison were conducted in cells cultures *in vitro* and do not necessarily reflect the *in vivo* situation, the high consistency of *cis-*eQTLs in whole blood and other tissues and in cell lines found here indicates that whole blood might be an informative tissue for an abundance of transcriptional regulatory relationships also in other tissues.

**Table 2:** Comparison of significant set of *cis*-eQTL with previously published results of eQTL studies conducted in different tissues.

First author	Cell line/Tissue	Publication Year	Sample Size	Number of RefSeq genes of significant *cis-*eQTL	Number of RefSeq genes annotated and significant in the present and the compared study	Overlap with RefSeq genes of our significant *cis-*eQTLs
**Validated results of the present study**						
Schramm	whole blood	2014	890	3,449		
**Databases for comparison**						
Fairfax [6]	monocytes	2011	283	7,468	1,764	51%
	B cells	2011	283	6,831	1,354	39%
Zeller [7]	monocytes	2011	1,490	2,477	1,620	70%
Schadt [8]	liver	2008	427	1,525	642	46%
Stranger [9]	LCLs	2007	90	412	229	61%
Fehrmann [2]	whole blood	2011	1,469	5,928	2,250	65%
Innocenti [10]	liver	2011	206	1,173	487	46%
Dixon [11]	LCLs	2007	400	727	373	57%
Sasayama [3]	whole blood	2013	76	308	171	68%
Hao [12]	lung	2012	1,111	9,138	2,252	65%

The proportion of overlap between the validated *cis-*results of the present study and those provided in the databases was calculated by dividing the number of RefSeq genes which were annotated and significant in the present and the compared study by the total number of RefSeq genes of significant *cis-*eQTLs in the present study (3,449).

### Functional properties of significant whole blood cis- and trans-eQTLs

In order to identify pathways that are significantly influenced by eQTLs in *cis* and *trans* in whole blood, we conducted pathway analyses using the Ingenuity Pathway Analyses (IPA) software. In the Ingenuity data base, 99% of confirmed significant *cis*-eQTLs (3,720 out of 3,768), and 97% of confirmed significant SNP-probe associations in *trans* (139 out of 144) were annotated. The analyses yielded two and ten significant canonical pathways with Benjamin-Hochberg false discovery rate below 2.28E-2 and 3.45E-2 for the *cis* and *trans* data set, respectively (Table 3).

**Table 3:** Results of the pathway analysis using Ingenuity Pathway Analysis.

Transcripts associated with cis-eQTL		
Significant Canonical Pathways	-log(B-H p-value)	Molecules
NAD Salvage Pathway II	1.89	NT5C3B,NT5C3A,ACP2,NMRK1,NT5E,NT5M,ACP1,NT5C2, ACP5,NMNAT3,ACPL2,ACPP
Glutathione Redox Reactions I	1.64	GSR,GSTT1,GPX3,MGST1,MGST2,GPX4, GPX7,PRDX6,MGST3,GSTK1

Transcripts associated with trans-eQTL
Significant Canonical Pathways	-log(B-H p-value)	Molecules
Allograft Rejection Signaling	4.38	HLA-DRB4,HLA-A,HLA-DRB3,HLA-DQA1,HLA-DQB1,HLA-DPB1
Cytotoxic T Lymphocyte-mediated Apoptosis of Target Cells	4.38	HLA-DRB4,HLA-A,HLA-DRB3,HLA-DQA1,HLA-DQB1,HLA-DPB1
OX40 Signaling Pathway	4.30	HLA-DRB4,HLA-A,HLA-DRB3,HLA-DQA1,HLA-DQB1,HLA-DPB1
Antigen Presentation Pathway	3.93	HLA-DRB4,HLA-A,HLA-DRB3,HLA-DQA1,HLA-DPB1
Cdc42 Signaling	3.33	HLA-DRB4,HLA-A,HLA-DRB3,HLA-DQA1,MYL4,HLA-DQB1,HLA-DPB1
Dendritic Cell Maturation	2.64	HLA-DRB4,HLA-A,HLA-DRB3,CREB1,HLA-DQA1,HLA-DQB1,IL37
Graft-versus-Host Disease Signaling	2.46	HLA-A,HLA-DQA1,HLA-DQB1,IL37
Crosstalk between Dendritic Cells and Natural Killer Cells	2.36	KIR2DL5B,HLA-DRB4,KIR2DL5A,HLA-A,HLA-DRB3
Communication between Innate and Adaptive Immune Cells	1.54	HLA-DRB4,HLA-A,HLA-DRB3,IL37
Autoimmune Thyroid Disease Signaling	1.46	HLA-A,HLA-DQA1,HLA-DQB1

Being part of the immune system major features of whole blood are connected to the immune response, a fact which was reflected in the results of pathway analyses of confirmed significant transcriptional regulatory relationships. Class I and II MHC genes were the driving force of all statistically significant canonical pathways identified using the *trans-*data set. A previous eQTL study conducted in monocytes and B cells also reported a key role of the class II MHC gene *HLA* in regulating gene expression in *trans*, however, only in monocytes, not in B cells [6], suggesting that some of the signal in whole blood tissue is probably monocyte derived. Likewise, in *cis*, a significant canonical pathway of the immune response was identified with ten genes coding for different glutathione peroxidases, reductases and transferases. All of them are involved in the glutathione redox reaction pathway which predominantly plays a role in the direct neutralization of reactive oxygen species (ROS). In addition to this, twelve genes (different acid phosphatases, nicotinamide nucleotide adenylyltransferase, nicotinamid riboside kinase, 5′,3′ nucleotidase, and different 5′ nucleotidases) were found to be part of the nicotinamide adenine dinucleotide (NAD) salvage pathway II, thus reflecting whole blood assignments involved in the basic maintenance of cellular functions.

### Connections with other genes, disease or trait phenotypes, and metabotypes

#### a) Master regulatory loci

**Figure 3:**
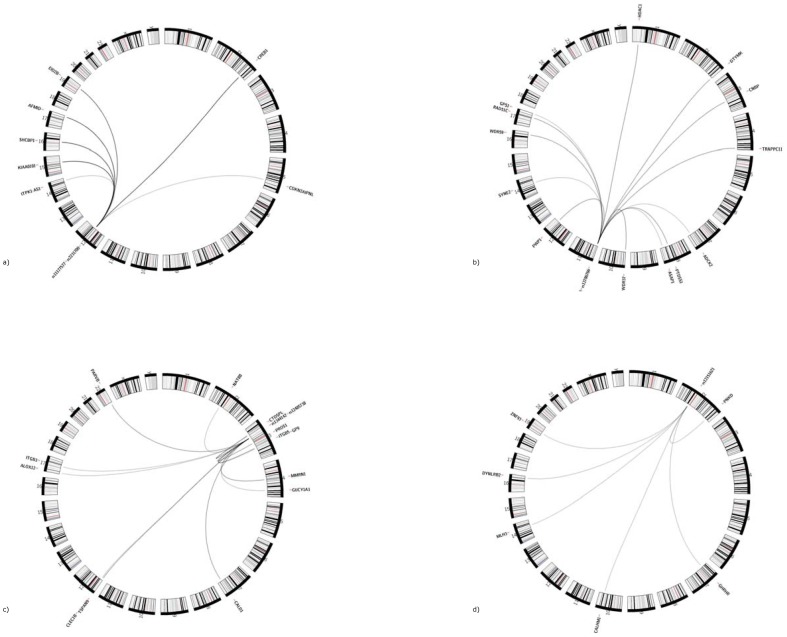
a–d): eQTLs with simultaneous impact on expression levels of at least five genes in *trans*. a) Chromosome 12. The eQTL was located upstream of *lysozyme* (*LYZ*), a gene residing on chromosome *12q15*. It is associated with expression levels of the seven transcripts *cAMP responsive element binding protein 1 (CREB1), SHC SH2-domain binding protein 1 (SHCBP1), arylformamidase (AFMID), KIAA0101, ITPK1 antisense RNA 1 (ITPK1-AS1), EP300 interacting inhibitor of differentiation 2B (EID2B)*, and *CDKN2A interacting protein N-terminal like (CDKN2AIPNL)*. b) Chromosome 11. The eQTL was found intronic of the *hemoglobin beta* (*HBB*) gene on chromosome *11p15.4* and was associated with the regulation of 13 genes distributed across the genome in *trans*: *PWP1 homolog (PWP1), phosphatidylserine synthase 1 (PTDSS1), CCHC-type zinc finger, nucleic acid binding protein (CNBP), trafficking protein particle complex 11 (TRAPPC11), histone deacetylase 1 (HDAC1), WD repeat domain 59 (WDR59), G protein pathway suppressor 1 (GPS1), ArfGAP with SH3 domain, ankyrin repeat and PH domain 1 (ASAP1), aarF domain containing kinase 2 (ADCK2), deoxythymidylate kinase (thymidylate kinase) (DTYMK), WD repeat domain 37 (WDR37), spectrin repeat containing, nuclear envelope 2 (SYNE2)*, and *RAD51 paralog C (RAD51C)*. c) Chromosome 3. The eQTL on chromosome 3 was located intronic of the *rho guanin nucleotid exchange factor 3 (ARHGEF3)* gene at *3p14.3*. We observed a significant impact on the regulation of twelve genes, *integrin beta 5 (ITGB5), platelet glycoprotein IX (GP9), carboxy-terminal domain, RNA polymerase II, polypeptide A small phosphatase-like (CTDSPL), protein S alpha (PROS1), guanylate cyclase soluble subunit alpha-3 (GUCY1A3)*, *caldesmon 1 (CALD1)*, *tetraspanin 9 (TSPAN9), arachidonate 12-lipoxygenase (ALOX12), parvin beta (PARVB), N-acetyltransferase 8B (NAT8B), multimerin 1 (MMRN1)*, and *C-type lectin domain family 1, member B (CLEC1B)*. d) Chromosome 2. The eQTL upstream of *atonal homolog 8 (ATOH8)* residing on chromosome 2p11.2 exerts simultaneous impact on expression levels of six genes: *paroxysmal nonkinesigenic dyskinesia (PNKD)* and *calcium homeostasis modulator 1 (CALHM1)*, *zink finger protein 93 (ZNF93), dynein, light chain, roadblock-type 2 (DYNLRB2), growth hormone-releasing hormone receptor (GHRHR)*, and *MutL-homolog 3(MLH3)*.

In our data, 21 *trans-*eQTL-SNPs were significantly associated with expression levels of two or more genes. Among those, we identified four “master regulatory loci” with significant simultaneous impact on expression levels of five or more genes in *trans* (Table 4). These loci reside on the chromosomes 12, 11, 3, and 2 (Figure 3 a–d). Their simultaneous impact on multiple expression levels in *trans* could be confirmed in the two replication samples.

**Table 4:** Master regulatory sites – eQTL-SNPs with simultaneous impact on the expression of at least five genes.

SNP*	chr.SNP	Gene of SNP	Probe_Id	Gene of probe	chr.gene	n	BETA	SE	Pvalue
rs12151621	2	N/A	ILMN_2282282	MLH3	14	888	0.190	0.014	1.31E-38
	2	N/A	ILMN_1679130	CALHM1	10	888	0.142	0.013	2.24E-25
	2	N/A	ILMN_1697317	DYNLRB2	16	888	0.139	0.013	1.12E-25
	2	N/A	ILMN_1652161	PNKD	2	888	0.141	0.012	1.89E-28
	2	N/A	ILMN_1724158	ZNF93	19	888	0.091	0.013	7.62E-13
	2	N/A	ILMN_1740186	GHRHR	7	888	0.078	0.009	6.93E-17
rs12485738	3	ARHGEF3	ILMN_1787919	PARVB	22	890	−0.136	0.019	2.77E-12
	3	ARHGEF3	ILMN_1729453	TSPAN9	12	890	−0.168	0.020	5.70E-16
	3	ARHGEF3	ILMN_1668374	ITGB5	3	890	−0.139	0.019	9.89E-13
	3	ARHGEF3	ILMN_2392189	CTDSPL	3	890	−0.130	0.017	9.16E-14
	3	ARHGEF3	ILMN_1743290	GP9	3	890	−0.179	0.022	1.82E-15
	3	ARHGEF3	ILMN_1730487	CALD1	7	890	−0.089	0.012	1.26E-13
	3	ARHGEF3	ILMN_1713731	ALOX12	17	890	−0.109	0.015	2.61E-12
	3	ARHGEF3	ILMN_1691264	NAT8B	2	890	−0.141	0.019	1.06E-13
	3	ARHGEF3	ILMN_1671928	PROS1	3	890	−0.162	0.018	7.82E-19
	3	ARHGEF3	ILMN_1808590	GUCY1A3	4	890	−0.107	0.015	3.58E-13
	3	ARHGEF3	ILMN_1660114	MMRN1	4	890	−0.089	0.011	1.23E-14
	3	ARHGEF3	ILMN_1745103	CLEC1B	12	890	−0.150	0.021	6.81E-13
rs10784774	12	N/A	ILMN_2334242	CREB1	2	889	0.190	0.012	5.33E-48
	12	N/A	ILMN_2182482	SHCBP1	16	889	0.189	0.010	3.29E-68
	12	N/A	ILMN_2095653	AFMID	17	889	−0.117	0.010	3.37E-30
	12	N/A	ILMN_2412521	KIAA0101	15	889	0.128	0.010	2.18E-35
	12	N/A	ILMN_2134381	ITPK1-AS1	14	889	0.093	0.013	2.02E-12
	12	N/A	ILMN_2051900	EID2B	19	889	0.150	0.013	6.00E-30
	12	N/A	ILMN_2130078	CDKN2AIPNL	5	889	0.072	0.010	2.74E-13
rs10742583	11	N/A	ILMN_1743049	PWP1	12	890	−0.142	0.011	1.20E-36
	11	N/A	ILMN_1688753	PTDSS1	8	890	0.239	0.012	5.79E-74
	11	N/A	ILMN_1769319	CNBP	3	890	0.114	0.010	1.02E-26
	11	N/A	ILMN_1752086	TRAPPC11	4	890	−0.075	0.009	9.90E-16
	11	N/A	ILMN_1727458	HDAC1	1	890	0.114	0.010	4.30E-28
	11	N/A	ILMN_1795428	WDR59	16	890	−0.094	0.009	8.94E-23
	11	N/A	ILMN_1795876	GPS1	17	890	0.065	0.008	4.36E-16
	11	N/A	ILMN_1690963	ASAP1	8	890	0.110	0.014	5.59E-14
	11	N/A	ILMN_1663132	ADCK2	7	890	0.094	0.012	2.55E-14
	11	N/A	ILMN_1716445	DTYMK	2	890	0.098	0.011	1.69E-19
	11	N/A	ILMN_1796464	WDR37	10	890	0.099	0.009	8.25E-25
	11	N/A	ILMN_1754579	SYNE2	14	890	0.067	0.008	5.92E-15
	11	N/A	ILMN_1695386	RAD51C	17	890	0.091	0.012	8.91E-15

*only the SNP which displayed strongest associations in the region is displayed.

Of note, all eQTLs with simultaneous impact on the regulation of five or more genes in *trans* were not significantly associated with the expression of a gene located in *cis* in our data. In contrast, one of our detected chromosomal regions, located upstream of *lysozyme* (*LYZ*) on chromosome *12q15* (Figure 3a) had previously been reported to exert effects in *cis* and *trans* in monocytes [6] pointing towards a monocyte specific effect in *cis* or – given a much smaller sample size of 283 subjects in the monocyte eQTL study - at least towards a much smaller effect in whole blood than in monocytes.

Our finding of merely significant effects in *trans* for the detected master regulatory sites implies that the impact of these regions on the activity of genes in *trans* in whole blood is stronger compared to an impact on the regulation of adjacent genes in *cis* if there is any on the latter at all. To analyze whether this is a random or rather an inherent and systematic feature, we had a closer look at all confirmed *trans*-eQTL-SNPs and found that the majority of them (58%) was not located in or near a protein coding chromosomal locus. Furthermore, about half of them (53%) did not exert a significant impact on a gene in *cis*. Thus, in the case of these SNPs, the effect on the regulation of genes in *trans* may not be explained by the modification of a gene in *cis* in our data.

In the past, it has been proposed that the spatial organization of DNA in the cell nucleus might be a key contributor to genomic function and that there might be “transcription factories” that engage inter-chromosomal interactions and form inter-chromosomal contacts [18]. In our data, one eQTL with simultaneous impact on multiple gene expression levels was located intronic of the *hemoglobin beta* (*HBB*) gene on chromosome *11p15.4* (Figure 3b). For the *HBB* mouse homologue a spatial network has been conjectured in a genome-wide analysis of transcriptional interactions using the mouse globin genes in erythroid tissues [19]. In this study, the *HBB* mouse homologue was associated with hundreds of active genes from nearly all chromosomes and it was presumed that the transcriptional regulation of the *HBB* gene involves a complex 3-dimensional network rather than factors acting on single genes in isolation [19]. Our data support evidence of a strong complex inter-chromosomal impact of the locus - but not necessarily of the *HBB* gene itself - with other genes and may serve as one example that indeed the spatial organization of DNA might be of relevance in this context.

#### b) Comparison with GWAS hits

Twenty-one percent of the genes detected in GWAS so far and recorded in the GWAS catalog (http://www.genome.gov/gwastudies, July, 18^th^, 2012, i.e. 3,508 genes reported in 1,310 publications) were identified to be eQTLs in our study. With respect to the identified eQTL dataset 746 of the identified eQTL-genes had been reported to be associated with clinical phenotypes. This finding might be relevant to learn more about previously reported associations to disease phenotypes and related traits.

**Figure 4:**
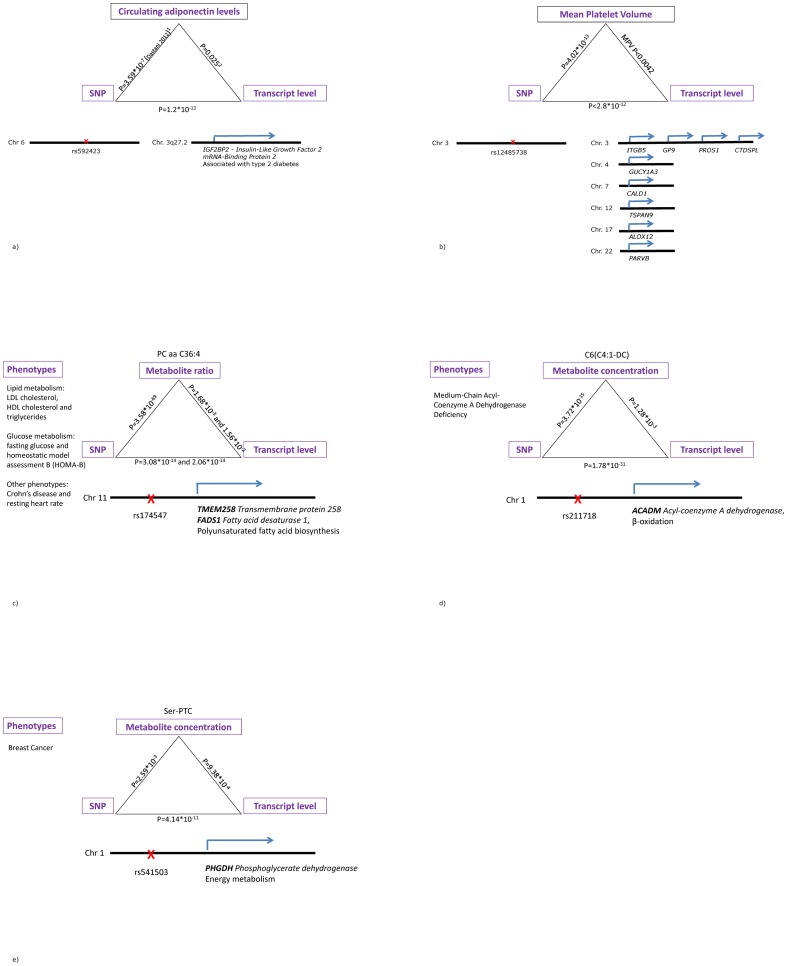
Triangular relationships between eQTL-SNPs, gene expression levels in *trans* and phenotypic traits. Figure 4a: Adiponectin. _1_ Measured in the fasting state. _2_ Measured 2-hours after an oral glucose load in oral glucose tolerance test. Figure 4b: Mean Platelet Volume (MPV). The association between SNP and mean platelet volume was assessed in 4,159 KORA S4 participants, those between gene expression levels and mean platelet volume in 889 participants of the KORA F4 study. Figure 4c–e: Correlation analyses combining genetic, metabolomics and transcriptomics data in 712 participants of the KORA F4 study.

For instance, SNP rs592423, residing in a gene desert on *6q24.1* was associated with adiponectin in a previous study [20]. In our study, this SNP is strongly associated with gene expression levels of probes mapping to the known type 2 diabetes susceptibility gene *insulin-like growth factor 2 mRNA-binding protein 2 (IGF2BP2)* residing in *trans* on chromosome *3q27.2*. In a subgroup of the KORA F4 discovery sample (N = 738), *IGF2BP2* expression levels were also significantly associated with adiponectin serum levels two hours after oral glucose tolerance test, so that a possible impact of rs592423 on adiponectin levels via the expression of *IGF2BP2* can be hypothesized (Figure 4a).

Several of those trait- or disease-relevant SNPs were identified to be eQTLs with simultaneous impact on the measured expression levels of multiple genes in our study. One example is rs199448 on chromosome *17q21*. In our data, this genomic region was associated with expression level changes of four genes in *trans* while in a GWAS it was found to play a role in Parkinson's disease [21].

Of particular biomedical and pharmaceutical interest are common risk variants with pleiotropic clinical effects. Notably, two of the SNPs with simultaneous impact on multiple gene expression levels also displayed such clinical pleiotropic effects. One example is the region on chromosome *12q15* (Figure 3a, Table 4), which had been found to affect height [22], pulmonary function decline [23], and response to diuretic therapy [24] in GWAS. Likewise, the chromosome *3p14.3* region with reported associations for creatinine levels [25], blood pressure [26], chronic kidney disease [27], and mean platelet volume [28], [29] was found to be a master regulatory site in our data (Figure 3c, Table 4). We assessed cross-associations between the lead SNP of this region, rs12485738, the twelve *trans*-eQTL genes, and mean platelet volume in 889 participants of the KORA F4 study. Thereby, we could provide evidence for a triangular relationship between the SNP, mean platelet volume and gene expression activity of nine out of the twelve annotated genes (Figure 4b).

#### c) Comparison with metQTLs

It has been shown in many studies that the metabolic phenotype (metabotype), as it can be determined in a sample of human blood, carries information on important biological processes, and that some metabolic traits represent intermediate phenotypes linking genetic and environmental factors to endpoints of complex disorders [30]. Therefore, in addition to the comparison with recorded results of GWAS, significant eQTLs were also compared to those results of a previously conducted GWAS of 163 metabolic traits measured in human blood from 1,809 participants of the KORA population with replication in 422 participants of the TwinsUK study [31]. In this study, multiple SNPs were identified to be *metabolomic quantitative trait loci (metQTLs)*, which means that they associate with metabolite concentrations or with the ratio between these concentrations as proxies for metabolic processes. In particular, in their study, Illig et al. detected 18 SNPs showing significant associations with more than 50 distinct metabolite concentrations and ratios between these concentrations (A full list of all associated metabolite concentrations and concentration ratios is provided in Table S2 of the original article [31]. Most of these SNPs were located in or near genes encoding enzymes with known functions involving the associated metabolite as products and/or substrates [30], [31]. Of those 18 metQTLs, six SNPs (rs174547, rs211718, rs8396, rs541503, rs272889, and rs964184) were congruent to eQTLs in our study (Table S3). Building on these results, we combined metabolomics and transcriptomics data, to identify triangular relationships between these SNPs and both the associated expression levels and the associated concentrations of metabolites or metabolite ratios. That way, we aimed to establish possible functional pathways. These analyses were conducted in a KORA F4 subpopulation (N = 717) in which both metabolomics as well as transcriptomics data were available. Significant triangular relationships between three SNPs, rs174547 (intronic of the *Fatty Acid Desaturase 1 (FADS1)* gene), rs211718 (upstream of *Acetyl-coenzyme A dehydrogenase (ACADM))*, and rs541503 (upstream of *Phosphoglycerate Dehydrogenase (PHGDH))*, the identified metabolic traits/ratio as well as transcript levels of genes in *cis* were identified (Figure 4c–e). For rs174547, we found similar associations with both SNP and metabolite ratio for transcript levels of the surrounding *FADS1* gene and those of the significantly correlated (P = 7.5E-9) proximate gene *Transmembrane Protein 258 (TREM258*, P-values: 2.06E-14 and 3.08E-14 for the SNP-transcript association and 1.68E-3 and 1.56E-2 for the transcript-metabolite ratio association for *TREM258* and *FADS1*, respectively).

All three SNPs had previously been reported to be associated with clinically relevant traits and endpoints such as cardiovascular disease, resting heart rate, Crohn's disease, and glucose as well as lipid metabolism for rs174547, medium-chain acyl-coenzyme A dehydrogenase deficiency for rs211718, and breast cancer for rs541503 (summarized in [31], [32]). Furthermore, all three SNPs were located in or near enzymes encoding genes with functions in human lipid metabolism. In the case of the adjacent genes of the three congruent SNPs those were polyunsaturated fatty acid biosynthesis *(FADS1)*, β-oxidation *(ACADM)*, and amino acid metabolism *(PHGDH)* [31]. In a subsequent study, Suhre et al. found a reflection of the function of the nearby gene in some of the associated metabolite concentrations. Thereby, they disclosed a possible mechanistic link between the genetic polymorphisms and metabolic products or processes via distinct activation of the adjacent genes [32]. In particular, for the three congruent SNPs, associations with a specific phosphatidylcholine ratio as a *FADS1* substrate/product pair ratio, a specific carnitine as a substrate of *ACADM*, and with serine concentrations for *PHGDH*, an enzyme which is thought to catalyze the first and rate-limiting step in the phosphorylated pathway of serine biosynthesis, are mentioned [32].

In the case of all three triangular relationships (Figure 4c–e), the expansion of these analyses to transcriptomics suggested that the modification of the enzyme encoding gene in *cis* is possibly an intermediate step connecting the three SNPs with the metabolic processes of polyunsaturated fatty acid biosynthesis, β-oxidation and amino acid metabolism (Figures 4c–e). Hence, altogether, these results very well support and complement evidence of a mechanistic link between SNP and human metabolism via modulation of the gene expression level of the enzyme encoding gene in *cis*.

With these examples, we showed that the combination of metabolomics and transcriptomics analyses provides a hypothesis-free approach and a promising way to pinpoint mechanistic links underlying associations between genetic variation and human metabolism.

### Conclusion

Including data of 890 subjects with validation in 1,818 subjects of two independent replication samples the present study represents one of the largest whole genome eQTL analyses studying effects in both *cis* and *trans* in whole blood samples in European populations so far. Within this study, different aspects were explored and several new insights were gained.

In spite of various systematic differences in the study design of the two replication samples (fasting versus non-fasting status of participants, time of blood collection, and different laboratory tools, namely, PAXgene™ versus Tempus™ tubes) replication overlaps of both replication samples were comparable (78% and 82%). Together with a total replication overlap of 91% in at least one of the samples this demonstrates a high robustness and reproducibility of genetically determined regulatory effects in whole blood. Consequently, whole blood eQTL studies provide a means for the discovery of biomarkers which are of clinical relevance for the perturbation of the system in a disease status.

In addition to this, we observed some cross-tissue similarities with *cis-*eQTLs found in other tissues and in cell lines (namely in primary monocytes, LCLs, B cells, lung, and liver). This finding is of relevance as it suggests that whole blood, the most convenient tissue for investigations, is a suitable surrogate tissue for *cis-*eQTL analysis conducted in the aforementioned tissues.

Pathway analysis for the significant eQTLs identified an enrichment of pathways involved in the development and the activity of the immune system and a central role of the HLA system and thus mainly reflected functional properties of whole blood assignment in the immune response.

Of the identified eQTL genes, 746 had been previously reported to be associated with clinically relevant traits or disease endpoints in humans. Thus, results of eQTL studies offer a valuable resource to investigate genetic mechanisms underlying gene-disease associations. Exemplarily, we showed that one SNP residing in a gene desert on chromosome *6q24.1* exerts its effect on adiponectin levels possibly via expression of a known type 2 diabetes susceptibility gene (*IGFBP2*). Particularly of biomedical and pharmaceutical interest are common risk variants with pleiotropic phenotypic effects. We identified four genetic regions which determine expression levels of multiple genes in *trans* and provided evidence of cross-associations between one of these genetic regions, expression levels of several genes in *trans* and mean platelet volume. Thus, we showed that eQTL analysis might provide a starting point for further functional studies and help to elucidate the colocalization of common risk variants which often connect diseases with little obvious mechanistic overlap. Finally, we exemplified that in extension to metabolomics data, eQTL studies provide a hypothesis-free approach to link genetic variation with human metabolism.

Taken together, the present study identified 3,847 eQTLs which were confirmed in at least one independent replication study in whole blood in a large European sample and provided evidence that these results offer a valuable resource for investigators studying the genetic architecture of regulatory pathways in whole blood. The high replication overlaps in spite of various systematic differences in the study design of one of the two replication samples demonstrated the robustness and reproducibility of genetically determined regulatory effects in whole blood, which was found to be an informative tissue for an abundance of transcriptional regulatory relationships also in other tissues.

## Materials and Methods

### Study populations

The KORA (Kooperative Gesundheitsforschung in der Region Augsburg - Cooperative Health Research in the Region of Augsburg) study is a series of independent population-based epidemiological surveys and follow-up studies of participants living in the region of Augsburg, Southern Germany. In the present study, we included 890 participants (448 males and 442 females aged 61 to 82 years [33]) of the KORA F4 study for whom genome-wide genotyping and gene expression data were available. KORA F4 (2006–2008) is the follow-up study of the KORA S4 survey (1999/2001). The standardized examinations applied in the survey (4261 participants) have been described in detail elsewhere [34]. A total of 3,080 subjects participated in the S4 follow-up examination (KORA F4) comprising individuals who, at that time, were aged 32–81 years. The study has been conducted according to the principles expressed in the Declaration of Helsinki. Written informed consent has been given by each participant. The study was reviewed and approved by the local ethical committee (Bayerische Landesärztekammer).

For the replication we used 976 samples of the Study of Health in Pomerania (SHIP). The Study of Health in Pomerania (SHIP) is a population-based project in West Pomerania, a region in the Northeast of Germany. For this project, the SHIP-TREND study was used. Baseline examinations for this study were carried out from 2008 to 2012. From the total population of West Pomerania comprising approximately 210,000 inhabitants, a stratified random sample of 8,826 adults was drawn. Stratification variables were age, sex, and city/county of residence. In total, 4,420 participants have been examined. Study design and sampling methods as well as genotyping and gene expression measurement and methods have been described elsewhere [35], [36]. The study followed the recommendations of the Declaration of Helsinki and was approved by the local ethical committee.

Furthermore, replication analysis was performed using data of the Estonian Genome Center Biobank, University of Tartu (EGCUT, www.biobank.ee), a population-based database which comprises the health, genealogical and genome data of currently more than 51,530 individuals. These individuals are aged 18 years or older and reflect closely the age distribution in the adult Estonian population. Participants of EGCUT were recruited by the general practitioners (GP) from GP offices, physicians from the hospitals or data collectors from EGCUT's patient recruitment offices. Each participant filled out a Computer Assisted Personal interview including personal data (place of birth, place(s) of living, nationality etc.), genealogical data (family history, three generations), educational and occupational history and lifestyle data (physical activity, dietary habits, smoking, alcohol consumption, and quality of life). Anthropometric and physiological measurements were also taken. The collection of blood samples and data is conducted according to the Estonian Gene Research Act and all participants have signed the broad informed consent.

### Gene expression and genotype data

In the KORA F4 study, the preparation of gene expression data on the Illumina HumanHT-12 v3 expression BeadChip was performed as described previously [36]. Gene expression data are available for download at ArrayExpress (E-MTAB-1708). All KORA F4 samples were genotyped using the Affymetrix 6.0 GeneChip. We limited our analysis to SNPs with a minor allele frequency >5% and a high genotyping quality (call rate >95%) and with respect to Hardy-Weinberg equilibrium (P_HWE_ > 1E-6). 616,941 SNPs fulfilled all these criteria. The preparation of the KORA F4 samples as well as of the SHIP-TREND and EGCUT samples is described in Table 1 to indicate the differences and similarities between the three studies.

### Gene expression analysis

A principal component analysis was conducted and different numbers of principal components (five to 100 in steps of five) were removed from the data by keeping the residuals in a linear model with expression as dependent and principal components as independent variables. Additionally, the uncorrected data and data corrected for age and sex were used. The software plink (http://pngu.mgh.harvard.edu/~purcell/plink/) was used to systematically calculate the association of all SNP-probe combinations with linear regression models using additive effects. The mean standard errors and betas were compared for different numbers of PCs.

Based on graphic analysis, we decided to correct for 55 PCs and 25 PCs for *cis*- and *trans*-analyses, respectively (Figure S3). This decision is in line with a previous study [2].

All 28,961 expression probes mapping to 18,606 RefSeq genes according to the annotation file of Schurmann et al. [36] were used for the analysis.

An eQTL was defined as being in *cis* if the SNP was located within 500 kb to the transcription start or end site of the respective gene (resulting in 8,308,176 possible SNP-probe combinations). The threshold of significance was defined on the basis of Bonferroni correction (6.02E-9 and 2.81E-12 for *cis-* and *trans-*eQTLs, respectively). For *cis-*eQTLs, only the SNP with the smallest p-value within the 500 kb window was selected, for *trans-*eQTLs we exclude all SNPs in high LD (>0.5) to a SNP regulating a gene in *cis*.

### Replication of cis and trans hits in SHIP-TREND and EGCUT

All significant SNP-probe combinations were also calculated in 976 SHIP-TREND and 842 EGCUT samples. The preparation of the SHIP-TREND and EGCUT samples is described in Table 1 to indicate differences and similarities between the three cohorts. For the replication either the same SNP or a proxy SNP was used. Linear models were adjusted for 50 (*cis*) and 25 (*trans*) principal components. Additionally, principal components that were associated with the SNP of interest were excluded from the analysis.

### Comparison of cis hits to results of eQTLs in other tissues and in cell lines

For the comparison of our results to eQTLs in other tissues and in cell lines, we downloaded Tables S2–S4 and S1 (*cis* hits in monocytes) of an eQTL study by Zeller et al. (2011) [7] in which the authors compared their *cis*-eQTLs in monocytes to published eQTLs in LCLs (Stranger et al, 2007 [9] and Dixon et al., 2007 [11]) as well as liver tissue (Schadt et al., 2008 [8]). Additionally, we compared our results to those listed in Table S2a from Hao et al. (2012) [12] who analyzed eQTLs in lung samples, Table S4 from Sasayama et al. (2013) [3] who used whole blood samples from Japanese individuals, Table S1 from Innocenti et al. (2011) [10] who analyzed liver samples, Table S1 from Fehrmann et al. (2011) [2] who used whole blood samples, and Table S1 from Fairfax et al. (2012) [6] who analyzed monocytes and B cells.

## Supporting Information

Figure S1 **Times of blood collection in the KORA F4 (red colored) discovery sample and the EGCUT replication sample (yellow colored) were assessed to determine their influence on eQTL results.**
(EPS)

Figure S2 **P-value plot for the comparison of results between KORA F4, SHIP-TREND, and EGCUT.**
(PDF)

Figure S3 **Effect of removing different numbers of principal components from expression data on the mean standard error and the number of significant** ***cis-*** **and** ***trans-*** **eQTLs.**
(PDF)

Table S1 **Confirmed significant** ***trans*** **-eQTLs.**
(XLS)

Table S2 **Confirmed significant** ***cis*** **-eQTLs.**
(XLS)

Table S3 **Results of cross-associations for congruent metQTL- and eQTL-SNPs.** 1 indicates p-values for the association between SNP and metabolic trait/ratio. 2 indicates p-values for the association between SNP and transcript level. 3 indicates p-values for the association between transcript level and metabolic trait/ratio. Congruent metQTL and eQTL-SNPs were identified by comparing eQTL results with metQTLs published by Illig et al. [31]. Cross-associations were calculated for a KORA F4 subpopulation for which genetic, metabolomics and transcriptomics data were available.
(XLS)
