# Supplementary figures and images for: Mapping the Genetic Architecture of Gene Regulation in Whole Blood
Source: PLoS One. 2014 Apr 16;9(4):e93844. doi: 10.1371/journal.pone.0093844 (PMC3989189; doi:10.1371/journal.pone.0093844)

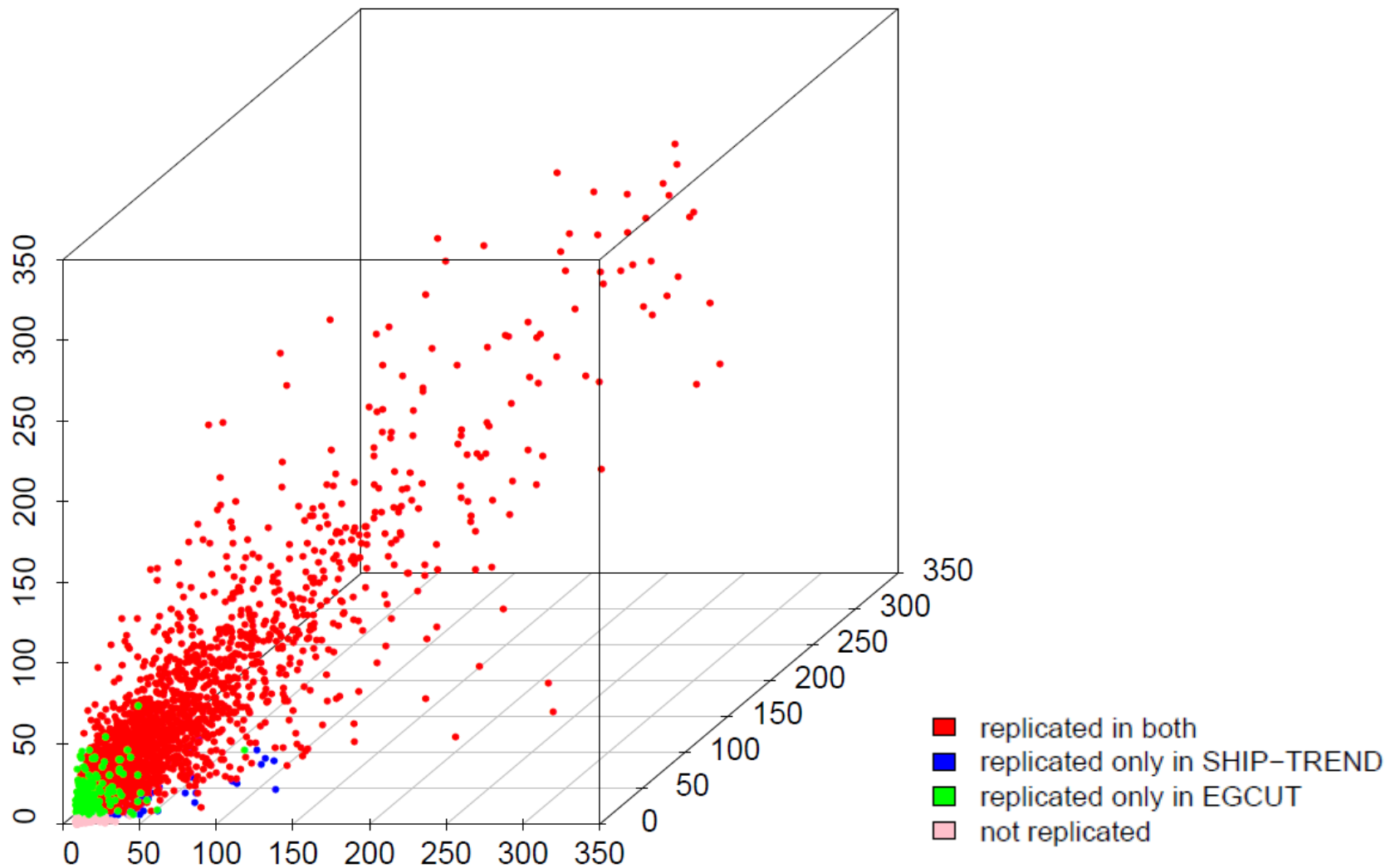

Suppl. Figure 2. X-, Y, and Z-axis present p-values for KORA F4, SHIP-TREND, and EGCUT, respectively.

Supplement: Figure S2 — P-value plot for the comparison of results between KORA F4, SHIP-TREND, and EGCUT. (PDF) [file pone.0093844.s002.pdf]

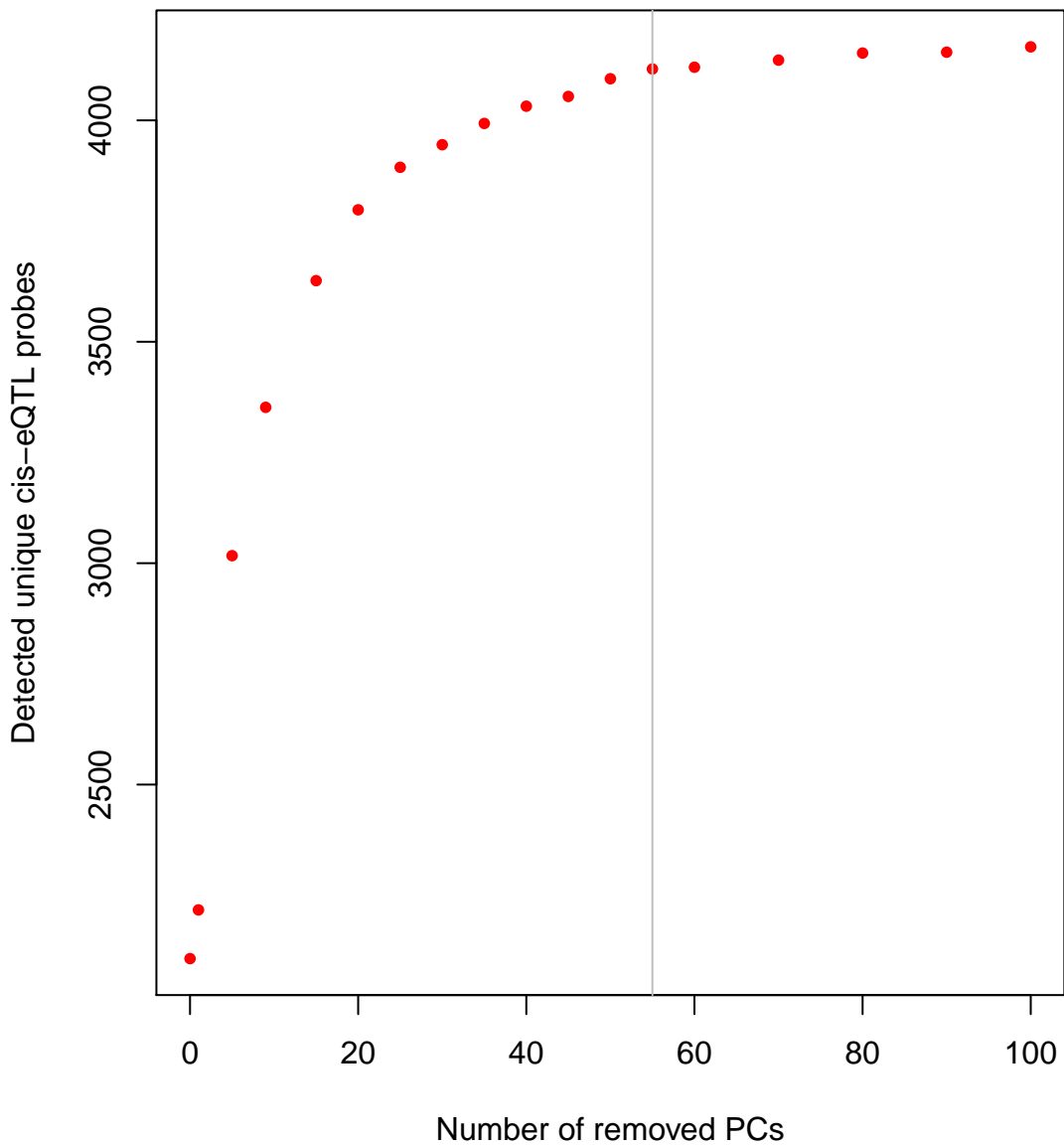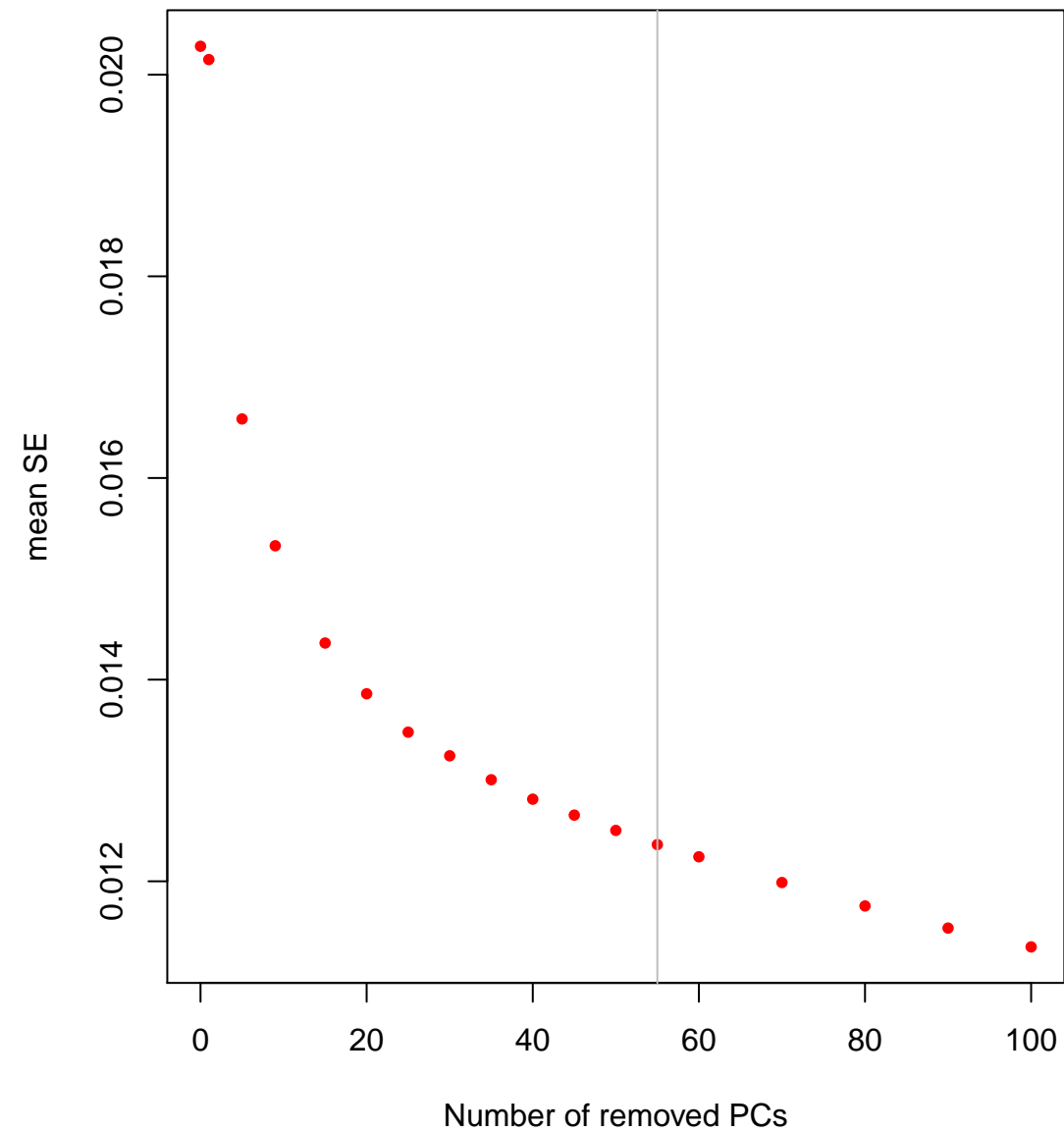

Detected unique trans-eQTL probes

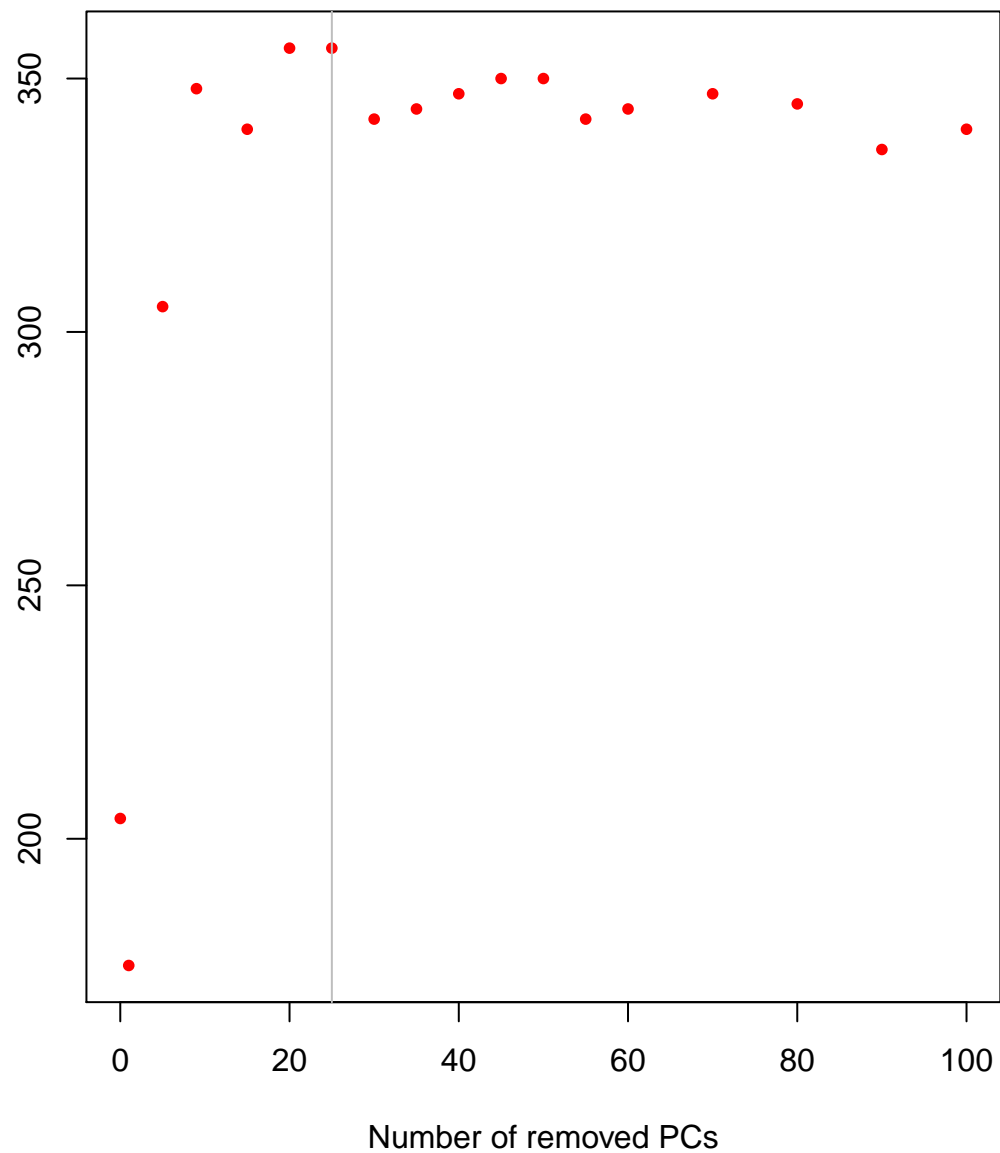

mean SE

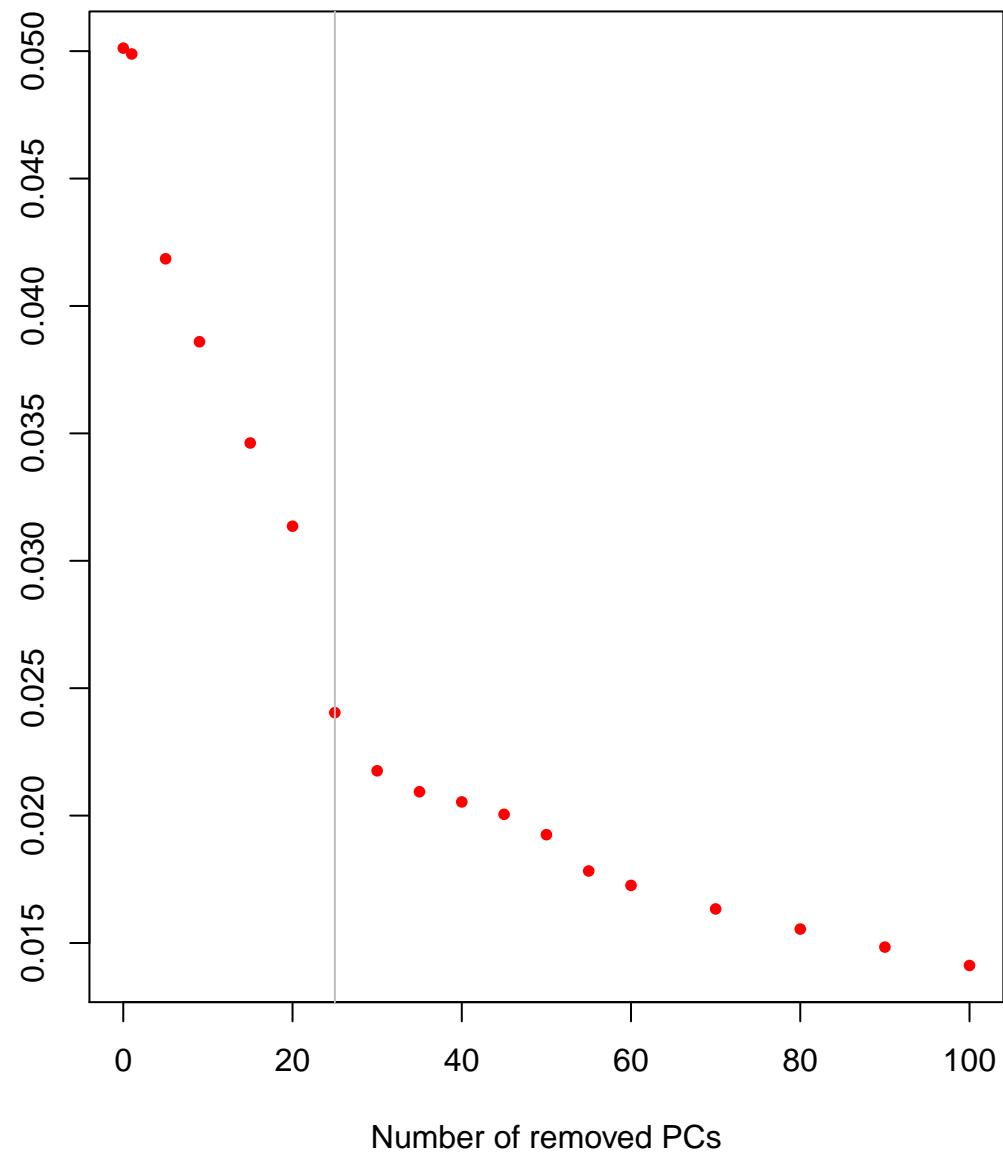

Supplement: Figure S3 — Effect of removing different numbers of principal components from expression data on the mean standard error and the number of significant cis- and trans- eQTLs. (PDF) [file pone.0093844.s003.pdf]
